# FoXA2 promotes esophageal squamous cell carcinoma progression by ZEB2 activation

**DOI:** 10.1186/s12957-021-02358-4

**Published:** 2021-09-22

**Authors:** Hanjing Gao, Zheng Yan, Haiyan Sun, Yanfang Chen

**Affiliations:** 1 Department of Radiation Oncology, Tianjin 4TH Centre Hospital, Tianjin, 300140 China; 2 Department of Integrated Chinese and Western Medicine, Tianjin Medical University Cancer Institute and Hospital, National Clinical Research Center for Cancer, Key Laboratory of Cancer Prevention and Therapy, Tianjin Clinical Research Center for Cancer, Tianjin, 300060 China; 3 Department of Radiation Oncology, Tianjin Medical University Second Hospital, No. 23, Pingjiang Road, Hexi District, Tianjin, 300211 China

**Keywords:** FOXA2, ESCC, Progression, ZEB2

## Abstract

**Background:**

It has been reported that Forkhead transcription family member (FOXA2) regulates esophageal squamous cell carcinoma (ESCC) progression. However, the specific mechanism, by which FOXA2 promotes ESCC malignant progression, remains unclear.

**Materials and methods:**

QRT-PCR and western blotting were applied to measure FOXA2 expression in ESCC tissues, while CCK-8 assay and Transwell assays were used to investigate the effect of FOXA2 on ESCC. Luciferase reporter assay, followed by fast chromatin immunoprecipitation (ChIP) assay, was used to study the relationship between FOXA2 and ZEB2.

**Results:**

FOXA2 was significantly increased in ESCC tissues, when compared to normal tissues. Moreover, high expression of FOXA2 was also found in ESCC cells. Knockdown of FOXA2 inhibited ESCC cell proliferation, invasion, and migration. Mechanically, FOXA2 was verified to regulate ZEB2 expression at transcription level. Moreover, ZEB2 reversed the inhibitory effect of FOXA2 on ESCC proliferation, invasion, and migration. The relationship between ZEB2 and FOXA2 in ESCC tissues was negative.

**Conclusions:**

These results indicate that FOXA2 plays a critical role in ESCC progression and may become a potential candidate target for ESCC treatment.

## Introduction

Esophageal cancer is one of the most common malignant tumors, with the incidence rate ranks third, and the cancer-related mortality rate ranks fourth in China [1, 2]. Among them, esophageal squamous cell carcinoma (ESCC) is the most histologic type [3–5]. Recently, radiotherapy and chemotherapy for the treatment of ESCC have received more and more attention, while targeted drug therapy and immunotherapy are still under development [6–8]. Therefore, a thorough understanding of the potential molecular mechanisms of ESCC and the search for new potential biomarkers are necessary for the early diagnosis and effective treatment of ESCC.

Forkhead box protein A2 (FOXA2), a member of the forkhead transcription factor family, plays an important regulatory role during embryonic liver organogenesis [9, 10]. Accumulating evidence indicate that the dysregulation of FOXA2 is related to the development of various cancers [11–13]. For instance, it has been reported that downregulation of FOXA2 promoted the ability of migration and invasion in lung cancer [14]. Moreover, FOXA2 functioned as a suppressor in hepatocellular carcinoma through PI3K/AKT signaling pathway [15]. Downregulation of FOXA2 enhanced the metastasis of pancreatic cancer by regulating the epithelial-to-mesenchymal transition (EMT) [16]. More importantly, David H. Wang et al. discovered that FOXA2 was expressed in esophageal adenocarcinoma but was not present in the normal esophageal squamous epithelium [17]. Therefore, we hypothesized that FOXA2 might be play important role in ESCC development. Here, we aimed to investigate the functional role of FOXA2 and its molecular mechanism in ESCC.

## Materials and methods

### Clinical samples

The tumor tissues and normal tissues were obtained from 30 patients diagnosed with ESCC at Tianjin 4TH Centre Hospital, Tianjin, China. All patients provided the written informed consent. All participants included in this study received neither radiotherapy nor chemotherapy before surgery. The collected tissues were stored at – 80 °C. The study protocols were approved by the ethics committee of the Tianjin 4TH Centre Hospital.

#### Inclusion criteria

ESCC patients, aged 18–75 years old, were confirmed by histology or cytology, and they were locally advanced unresectable or with distant metastasis. The advanced or metastatic ESCC patients have failed first-line chemotherapy, have at least one measurable lesion, and meet the RECIST1.1 standard. The body is in good condition, ECOG: 0 to 1.

#### Exclusion criteria

The patients have been diagnosed with other malignant tumors within 5 years, have central nervous system metastasis, have an any active autoimmune disease or a history of autoimmune disease, and have uncontrolled clinical symptoms or diseases of the heart.

### Cell culture and cell transfection

The ESCC cell lines ECA109 (MZ-2019) and KYSE-140 (MZ-2441), and human esophageal epithelial cells Het-1A were purchased from Mingzhou Biological Technology Co., Ltd (Zhejiang, China). The cells were cultured in RPMI1640 medium containing 10% FBS and incubated at 37 °C under a 5% CO2 humidified atmosphere. All transfection procedures were performed using Lipofectamine 2000™ reagent (Invitrogen), in line with the manufacturer’s instructions. The small interfering RNA (siRNA) was transfected into ESCC cells and incubated for 24 h at 37 °C.

### RNA extraction and quantitative real-time PCR (qRT-PCR)

Total RNA was isolated from ESCC tissue samples with Trizol reagent (Invitrogen). The RNA was reverse-transcribed to cDNA using Transcriptor First Strand cDNA Synthesis kit (Roche according to the manufacturer’s instructions. QRT-PCR was performed using the 7500 Real-Time PCR system (Applied Biosystems). FOXA2 expression was normalized to that of GAPDH, using the 2^−ΔΔCt^ method. The primers were as follows: FOXA2-F: 5′-TGCCATGCACTCGGCTTCCA-3′, FOXA2-R: 5′-CCCAGGCCGGCGTTCATGTT-3′; GAPDH-F, 5′-TCATTGACCTCAACTA CATGGTTT-3′, GAPDH-R: 5′-GAAGATGGTGATGGGATTTC-3′. ZEB2-F: 5′-CA CACACATACACAGAAAGGA-3′, ZEB2-R: 5′- ATAACAGGAGGCATAGCATT-3′.

### Western blotting

Total proteins were isolated from the ESCC using radio immunoprecipitation assay (RIPA) lysis buffer, and the concentrations were determined by BCA method. Equal amounts of protein (50 μg) were separated by 10% SDS-PAGE and then transferred to PVDF membrane. After blocking by incubation with 5% skim milk for 2 h, the membranes were incubated with primary antibodies such as FOXA2, ZEB2, and GAPDH. Antibody binding was detected with horseradish peroxidase-conjugated secondary antibodies followed by visualizing using enhanced ECL reagent. The loading control used was GADPH.

### Cell counting kit-8 (CCK-8) assay

ESCC cells were seeded in 96-well plates and cultured for 24 h at 37 °C under 5% CO2. At 24, 48, 72, and 96 h, 10 μL CCK-8 solution was added to ESCC cells, followed by incubation for another 2 h. The absorbance was analyzed at 450 nm using a microplate reader.

### Transwell assays

The migration and invasion cells were measured by transwell chambers with or without Matrigel-coated. ESCC cells were seeded in the top chamber, and 200 μL RPMI-1640 medium containing 20 % FBS medium was added into the lower chamber. After incubation for 24 h, cells that migrated or invaded through the membranes were collected and stained with 0.1% crystal violet. Images of the migrating cells or invading cells were photographed under a microscope.

### Chromatin immunoprecipitation (ChIP) assay

The cell lysis buffer containing the protease inhibitor (Sigma-Aldrich Co.) was used for lysis ESCC cells. Then, the corresponding primary antibodies were added and incubated at 4 °C overnight, followed by protein G magnetic beads for 2 h. After the immunoprecipitation reaction, the SDS buffer was added and boiled for 5 min. The complex was subsequently detected by western blotting analysis.

### Luciferase reporter assay

The fragment of the ZEB2 was cloned into the pGL3 promoter vector to form ZEB2-WT or ZEB2-MUT vector. The luciferase reporter vectors and siRNAs were co-transfected into ESCC cells using Lipofectamine 2000 Reagent. After transfection for 48 h, the relative luciferase activity was measured by Dual-Luciferase Reporter Assay System.

### Statistical analysis

Data are presented as the mean ± standard (SD) in triplicate measurements. The statistical analyses and graphs were performed using the SPSS 17.0 software and the Prism 6 GraphPad, respectively. Student’s *t* test was used for two-group comparisons, while one-way ANOVA and Tukey’s post hoc test were used for comparisons among multiple groups. The correlation between FOXA2 and ZEB2 was measured by Pearson correlation coefficients. A value of *P* < 0.05 was considered as statistically significant.

## Results

### FOXA2 was highly expressed in human ESCC tissues and cells

**Fig. 1:**
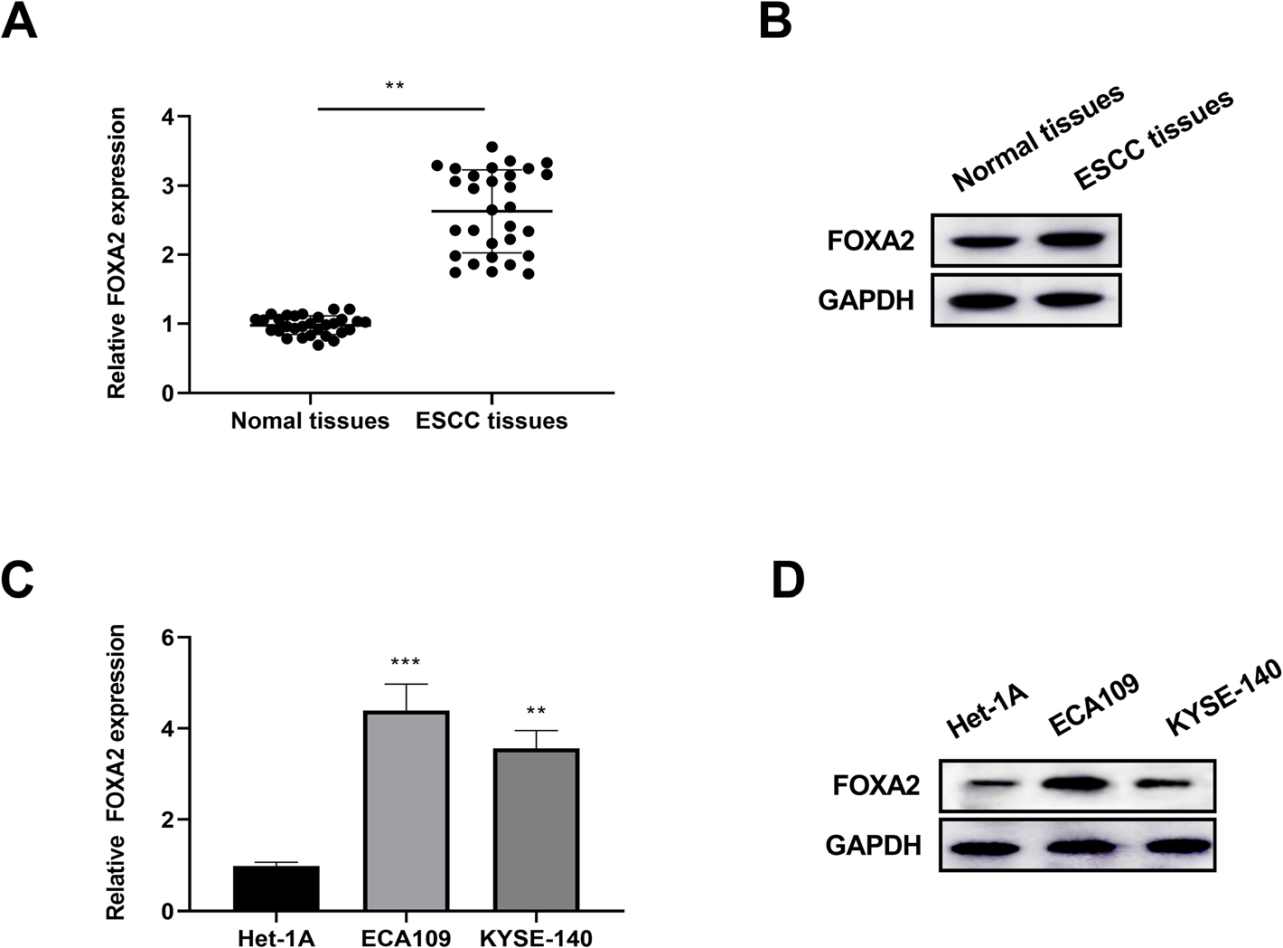
FOXA2 was increased in ESCC tissues and cells. A qRT-PCR and B western blotting were utilized to examine the mRNA level and protein level of FOXA2 extracted from ESCC tissues (*n* = 30) and adjacent non-cancer tissues (*n* = 30). C The expression of FOXA2 was analyzed in ESCC cell lines (ECA109 and YESE-140) and human esophageal epithelial cell line Het-1A by qRT-PCR and D western blotting. The data were repeated three times. **P < 0.01, ***P < 0.001

The expression of FOXA2 in ESCC tissues was detected by qRT-PCR and western blotting. Results showed that FOXA2 was significantly increased in human ESCC tissues, when compared to normal adjacent tissues, indicating that FOXA2 might exhibit a critical role in the progression of ESCC (Fig. 1A, B). Moreover, high expression of FOXA2 was statistically associated with TNM stage, distant metastasis, and relapse (Table 1). Furthermore, the expression of FOXA2 in ESCC cells was detected by qRT-PCR, followed by western blotting. Results showed that FOXA2 was significantly upregulated in ESCC cell lines versus to that in Het-1A cells (Fig. 1C, D). Taken together, FOXA2 was highly expressed in ESCC tissues and cells.

**Table 1:** Correlation between the clinicopathologic characteristics and FOXA2 expression in ESCC

Item	Cases (*n* = 30)	FOXA2		*P*-value
		Low (*n* = 14)	High (*n* = 16)	
**Age (years)**				0.732
< 55	14	7	7	
≥ 55	16	7	9	
**Gender**				0.961
Male	17	8	9	
Female	13	6	7	
**Tumor size**				0.028*
< 5 cm	15	4	11	
≥ 5 cm	15	10	5	
**Lesion location**				0.491
Upper/middle	17	7	10	
Lower	13	7	6	
**TNM stage**				0.024*
I–II	13	3	10	
III–IV	17	11	6	
**T classification**				0.003*
T1–T2	13	2	11	
T3–T4	17	12	5	
**N classification**				0.017*
N0	11	2	9	
N1–N3	19	12	7	
**Distant metastasis**				0.038*
M0	10	2	8	
M1	20	12	8	
**Relapse**				0.024*
Negative	8	1	7	
Positive	22	13	9	

Statistical analyses were performed by the *χ*2 test

*P < 0.05 was considered significant

### Silence of FOXA2 suppressed ESCC cell proliferation

**Fig. 2:**
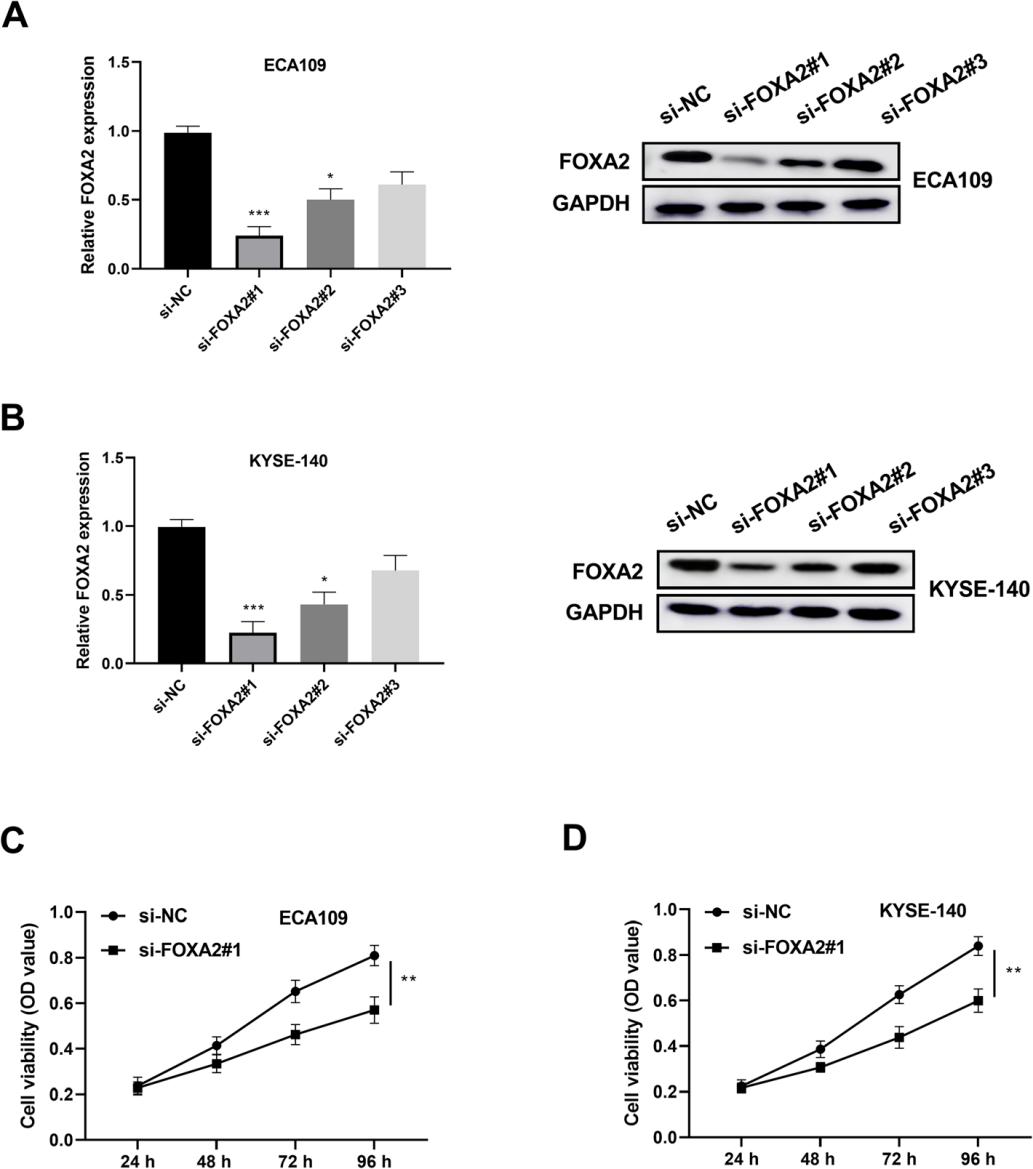
Knockdown of FOXA2 inhibited ESCC cells proliferation. **A** FOXA2 expression was detected in ECA109 and **B** YESE-140 cells after transfection with FOXA2 siRNA by using qRT-PCR and western blotting assays. The cells transfection with scramble siRNAs was used as the control group. **C** Cell viability was measured by CCK-8 assay in ECA109 cells transfection with si-FOXA2#1. **D** Cell viability was detected by CCK-8 assay in YESE-140 cells transfection with si-FOXA2#1. All experiments were repeated in three times. *P < 0.05, **P < 0.01, ***P < 0.001

CCK-8 assay was applied to examine the impact of FOXA2 on ESCC proliferation. ECA109 cells were transfected with FOXA2 siRNA and the transfection efficiency was determined by western blotting and qRT-PCR assays. As Fig. 2A, B shown, FOXA2 was reduced significantly, which was achieved by FOXA2 siRNA using qRT-PCR and western blotting assays. si-FOXA2#1 was selected for further experiments for its stronger inhibitory effects compared with si-FOXA2#2 or si-FOXA2#3. Results from CCK-8 assay discovered that FOXA2 knockdown suppressed the viability of ECA109 cells (Fig. 2C) and KYSE-140 cells (Fig. 2D). These data showed that knockdown of FOXA2 suppressed ESCC cell proliferation.

### Silence of FOXA2 inhibited ESCC cell migration and invasion

**Fig. 3:**
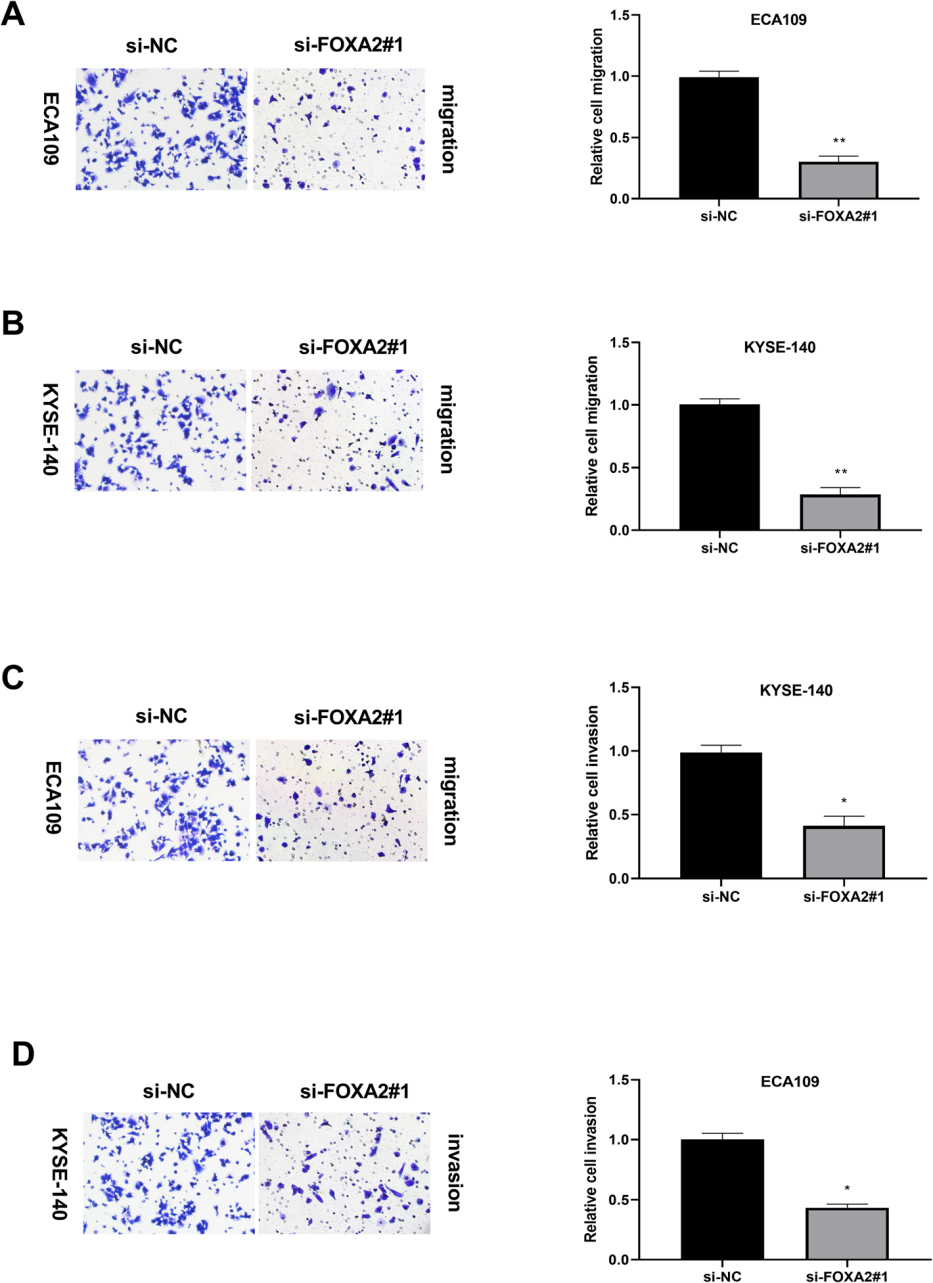
Silence of FOXA2 suppressed ESCC cells migration and invasion. **A** The effect of si-FOXA2#1 on the migration of ECA109 cells and **B** YESE-140 cells was detected by transwell assay, and the representative images and statistical analysis were shown. **C** The effect of si-FOXA2#1 on the invasion of ECA109 cells and **D** YESE-140 cells was measured by transwell assay, and the representative images and statistical analysis were represented.*P < 0.05, **P < 0.01

The effect of FOXA2 knockdown on ESCC cells migration and invasion was evaluated by transwell assays. Results showed that downregulation of FOXA2 decreased the ability of migration in ESCC cells in comparison with that of the control groups (Fig. 3A, B). Similarly, knockdown of FOXA2 suppressed the invasion ability of ESCC cells (Fig. 3C, D).

### FOXA2 repressed ZEB2 expression transcriptionally

**Fig. 4:**
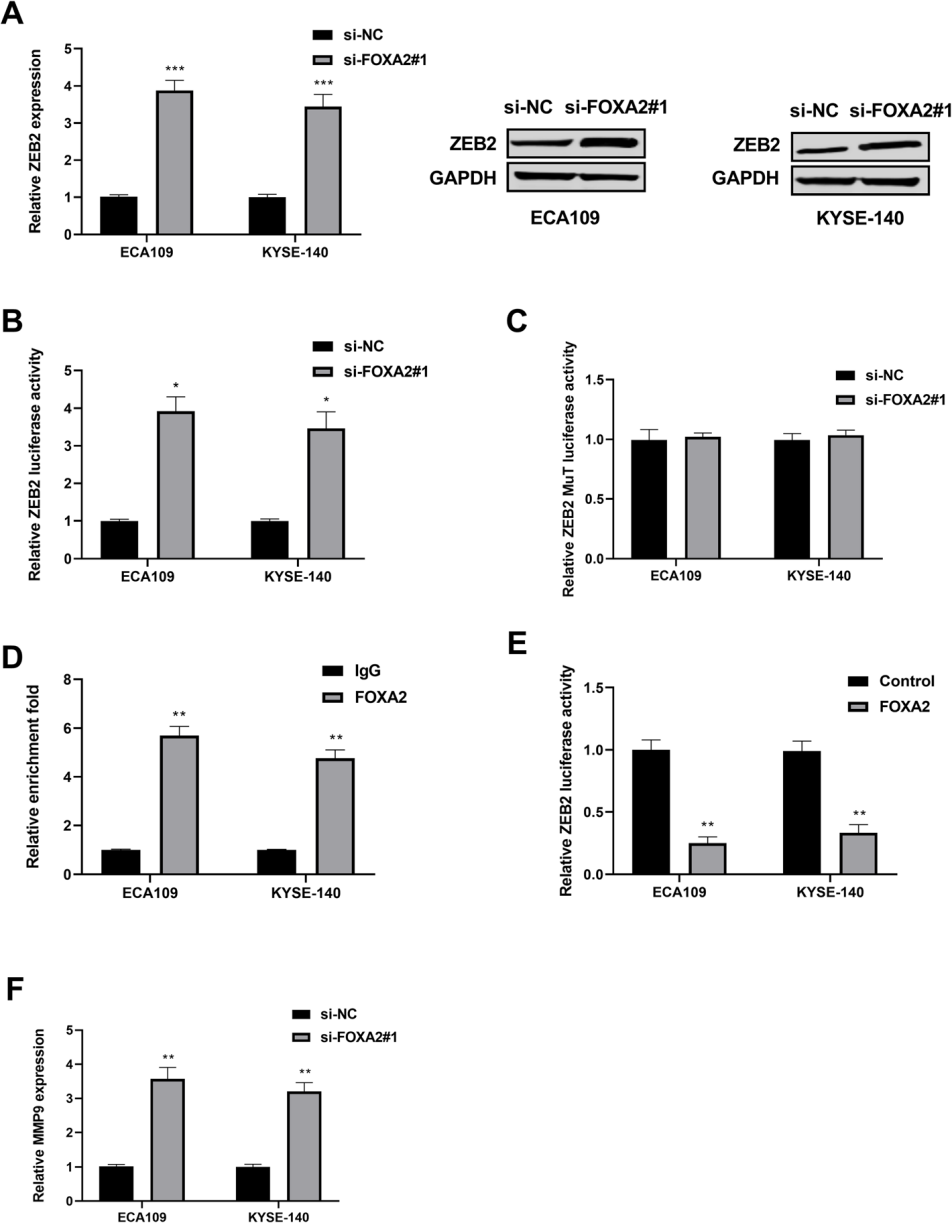
FOXA2 repressed ZEB2 expression transcriptionally. **A** ZEB2 expression was measured in ECA109 and YESE-140 cells transfection with si-FOXA2#1 by qRT-PCR and western blotting assays. **B** The luciferase activity of pGL3-ZEB2 reporter vector was detected in ECA109 and YESE-140 cells transfection with si-FOXA2#1. **C** The luciferase activity of pGL3-ZEB2 mutant vector was detected in ECA109 and YESE-140 cells transfection with si-FOXA2#1. **D** The binding of FOXA2 on the promoter of ZEB2 was confirmed by ChIP assay in ECA109 and YESE-140 cells. **E** The luciferase activity of pGL3-ZEB2 vector was detected in ECA109 and YESE-140 cells transfection with FOXA2 plasmids. **F** MMP9 expression was analyzed in ECA109 and YESE-140 cells transfection with si-FOXA2#1. *P < 0.05, **P < 0.01, ***P < 0.001

Previous studies have reported that ZEB2 plays an important regulatory role in the process of tumor metastasis. This present study explored whether ZEB2 was involved in the progression of ESCC modulated by FOXA2. Firstly, the expression of ZEB2 in ESCC cells after transfection with si-FOXA2#1 was measured. The findings showed that ZEB2 was significantly increased in both ECA109 and KYSE-140 cells transfection with si-FOXA2#1 versus to that in the control group (Fig. 4A). Subsequently, the underlying mechanism of FOXA2-mediated ZEB2 regulation and the effect of FOXA2 on ZEB2 transcription were investigated using a luciferase reporter assay. As Fig. 4B presented, downregulation of FOXA2 increased the luciferase activity of ZEB2-WT. However, there was no impact discovered on ZEB2-MuT (Fig. 4C). Results from ChIP assay demonstrated that FOXA2 directly binds to the ZEB2 promoter (Fig. 4D), and confirmed that up-regulation of FOXA2 reduced the expression of ZEB2 (Fig. 4E). Since MMP9 is a downstream target of ZEB2, the impact of FOXA2 on MMP9 expression was tested as well. Results showed that silence of FOXA2 significantly promoted MMP9 expression (Fig. 4F).

### ZEB2 reversed the inhibitory effect of FOXA2 on ESCC progression

**Fig. 5:**
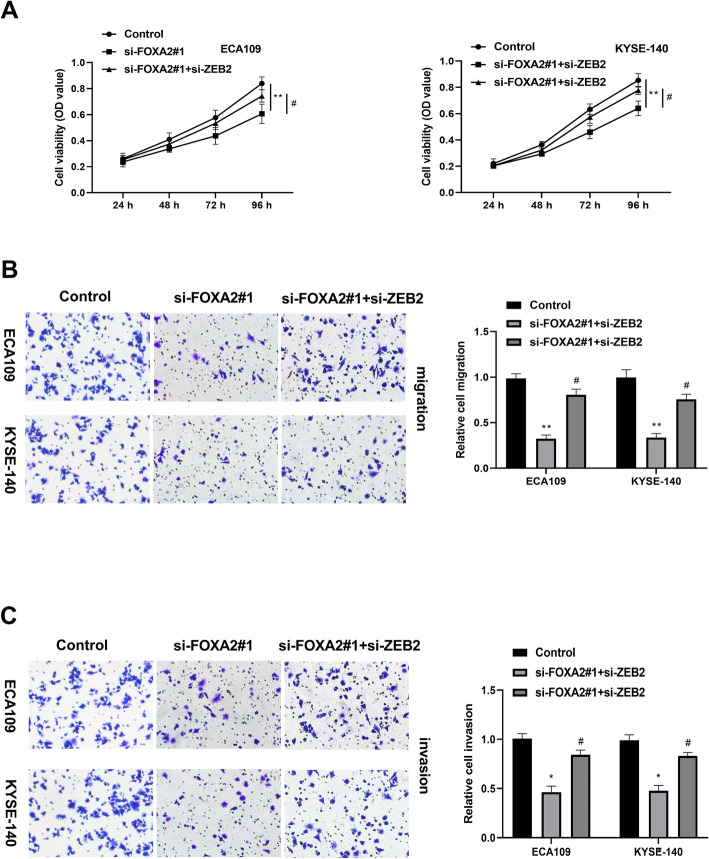
Silence of ZEB2 reversed the inhibitory effect of si-FOXA2#1 on ESCC progression. **A** Cell viability was measured in ECA109 and YESE-140 cells transfection with si-FOXA2#1 or combined with si-ZEB2. **B** Cell migration was measured in ECA109 and YESE-140 cells transfection with si-FOXA2#1, or combined with si-ZEB2. **C** Cell invasion was detected in ECA109 and YESE-140 cells transfection with si-FOXA2#1, or combined with si-ZEB2. *P < 0.05, **P < 0.01; #*P* < 0.05

To investigate whether ZEB2 inhibition takes part in the inhibitory effect of FOXA2, ECA109 and KYSE-140 cells were transfected with si-FOXA2#1, combined with si-ZEB2. As shown in Fig. 5A, the inhibitory effect of si-FOXA2#1 on ECA109 and KYSE-140 cells proliferation was partially overturned by si-ZEB2. Moreover, the migration ability of ECA109 and KYSE-140 cells in si-FOXA2#1 + si-ZEB2 group was remarkably increased in comparison with si-FOXA2#1 group (Fig. 5B). Similarly, the invasion ability reduced by si-FOXA2#1 was partly increased by co-transfection of si-FOXA2#1 and si-ZEB2 (Fig. 5C).

### Negative correlation of FOXA2 with ZEB2

**Fig. 6:**
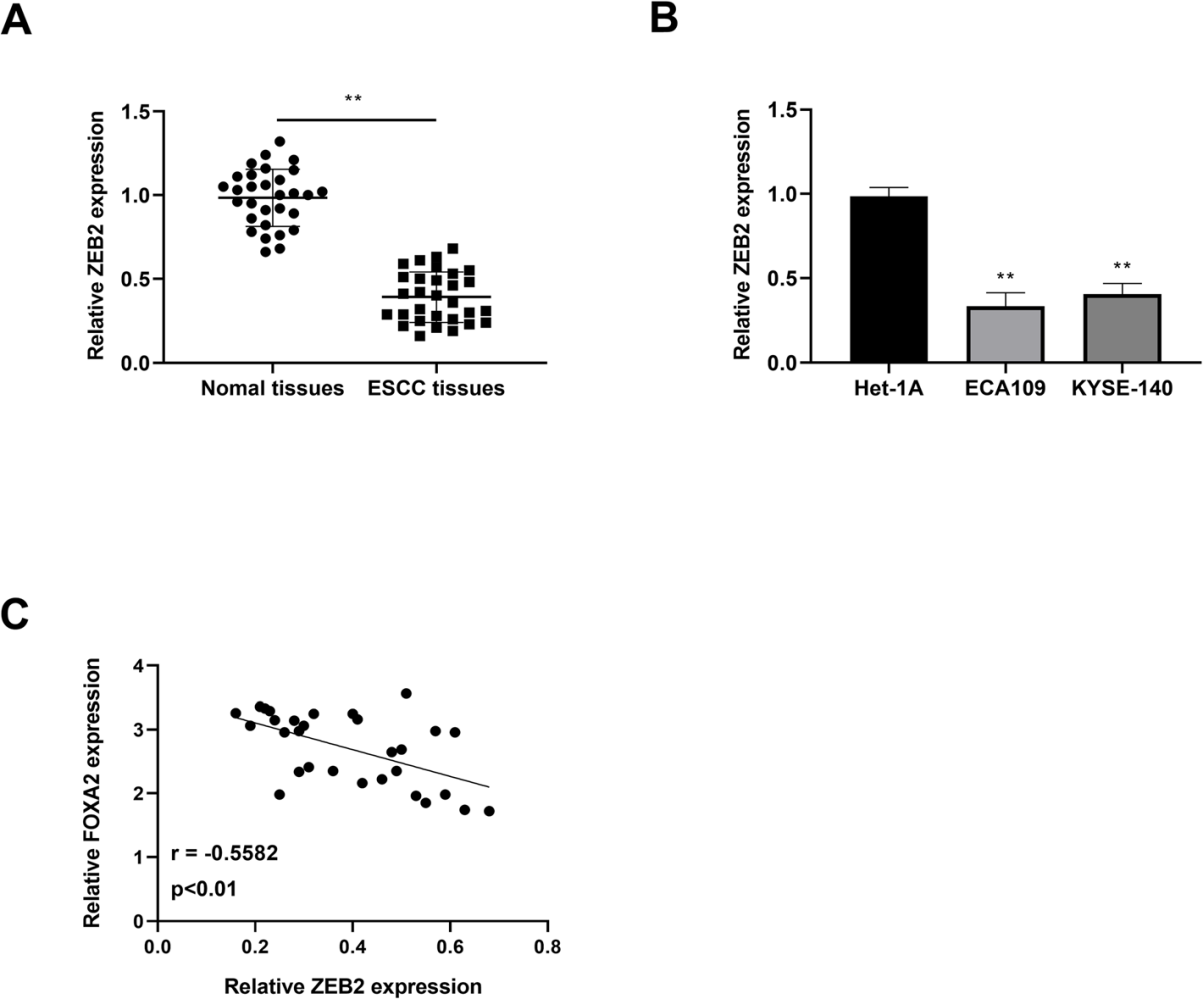
The negative correlation between ZEB2 and FOXA2. **A** qRT-PCR was utilized to examine the mRNA level of ZEB2 extracted from ESCC tissues (*n* = 30) and adjacent non-cancer tissues (*n* = 30). **B** The expression of ZEB2 was analyzed in ESCC cell lines (ECA109 and YESE-140) and human esophageal epithelial cell line Het-1A by qRT-PCR. **C** The correlation between FOXA2 and ZEB2 was detected in ESCC tissues. **P < 0.01

The relationship between FOXA2 and ZEB2 was then detected in ESCC. QRT-PCR was carried out to examine ZEB2 expression in ESCC tissues. The findings displayed that ZEB2 was decreased in comparison with the normal tissues (Fig. 6A). Similar to the results, ZEB2 expression was lower in ESCC cells than that in the normal cells (Fig. 6B). Furthermore, regression analysis results showed that the correlation of FOXA2 expression and ZEB2 expression was negative in ESCC tissues (Fig. 6C).

## Discussion

The incidence of ESCC has risen from 9th to 7th among all malignant tumors [18]. Although significant progress has been made in the treatment of ESCC, the prognosis of ESCC patients is still poor [19]. Therefore, it is important to identify ESCC-related biomarkers and explore the pathogenesis of ESCC. This present study discovered that FOXA2 knockdown inhibited ESCC tumorigenesis. This is the first reported showing that FOXA2 modulated ESCC progression by activating ZEB2, suggesting that FOXA2 was a promising therapeutic target for ESCC.

It has been reported that FOXA2 plays an important role in tumors development [17, 20, 21]. Wang et al. disclosed that, as an oncogene, FOXA2 shows a promotion effect on the proliferation, migration, and invasion of colon cancer [22]. An in vitro study showed an increased expression of FOXA2 in breast cancer, and that it positively regulated cell proliferation [23]. However, in lung cancer, pancreatic cancer, and colorectal cancer, FOXA2 was downregulated in tumor tissues, when compared to non-tumor tissues [24–26]. In the present study, it was observed that FOXA2 was highly expressed in ESCC and its high expression was associated with TNM stage, distant metastasis, and relapse. In addition, FOXA2 was upregulated in esophageal adenocarcinoma and worked as a potential indicator in the tumor development [17, 27, 28]. These results were consistent with our findings that FOXA2 knockdown suppressed ESCC proliferation, migration, and invasion.

To explore the molecular mechanisms underlying ESCC, luciferase reporter assay and ChIP assay were applied to confirm the target genes of FOXA2. Previous studies have demonstrated that the abnormal activation of EMT plays a critical role in the progress of tumor migration and invasion [29, 30]. ZEB2 has been identified as an important transcription factor in the epithelial-mesenchymal transition (EMT) process [31]. For example, Li et al. displayed that ZEB2 was involved in the metastasis of HCC [32]. In another study, knockdown of ZEB2 suppressed the migration and invasion of esophageal cancer [33]. As an EMT regulator, ZEB2 medicated the metastasis of breast cancer [34]. This present study showed a decreased expression of ZEB2, and the downregulation of ZEB2 rescued ESCC cell proliferation, migration, and invasion reduced by FOXA2 knockdown.

In conclusion, the experimental data demonstrated that FOXA2 was highly expressed in ESCC tissues and cells. Knockdown of FOXA2 inhibited ESCC proliferation, migration, and invasion via ZEB2 activation.

## Data Availability

All data in this study were obtained from public databases.
